# The Effect of Cold Exposure on Cognitive Performance in Healthy Adults: A Systematic Review

**DOI:** 10.3390/ijerph18189725

**Published:** 2021-09-15

**Authors:** Marika Falla, Alessandro Micarelli, Katharina Hüfner, Giacomo Strapazzon

**Affiliations:** 1 Center for Mind/Brain Sciences, CIMeC, University of Trento, 38068 Rovereto, Italy; 2 Institute of Mountain Emergency Medicine, Eurac Research, 39100 Bolzano, Italy; alessandro.micarelli@eurac.edu; 3 ITER Center of Balance and Rehabilitation Research (ICBRR), 02032 Rome, Italy; 4 Department of Psychiatry, Psychotherapy and Psychosomatics, University Hospital for Psychiatry II, Innsbruck Medical University, 6020 Innsbruck, Austria; katharina.huefner@tirol-kliniken.at

**Keywords:** cognition, cold, attention, memory, processing speed, executive function, hypothermia

## Abstract

Several aspects of cognition can be affected after cold exposure, but contradictory results have been reported regarding affected cognitive domains. The aim of the current systematic review was to evaluate the effects of specific cold exposure on cognitive performance in healthy subjects. A systematic search was performed using MEDLINE (through PubMed), EMBASE (Scopus) and PsycINFO databases according to the Preferred Reporting Items for Systematic Reviews and Meta-Analyses guidelines. Inclusion criteria were healthy subjects exposed to a cold environment (either simulated or not) and cognitive performance related to cold exposure with an experimental design. The literature search identified 18 studies, eight studies investigated the effect of cold air exposure and ten the effect of cold water immersion on cognitive performance of healthy subjects. There were several differences among the studies (environmental temperature reached, time of exposure, timing, and type of cognitive test administration). Cold exposure induced in most of the experimental settings (15 of 18) an impairment of CP even before accidental hypothermia was established. The most investigated and affected cognitive domains were attention and processing speed, executive function, and memory. Gender differences and effects of repeated exposure and possible acclimation on cognitive performance need further studies to be confirmed.

## 1. Introduction

The regulation of body temperature is activated in a cold environment. The primary responses to cold comprise shivering, inhibition of sweating, and skin vasoconstriction which allow the maintenance of a normal core body temperature of 37 °C. These are regulated by nervous feedback mechanisms mediated through temperature regulating centers located in the hypothalamus, temperature receptors in the skin, and in a few specific deep tissues of the body (e.g., spinal cord, abdominal viscera) [1,2]. The hypothalamus induces the stimulation of the sympathetic nervous system (SNS) and the primary motor center for shivering (PMC). The SNS induces skin vasoconstriction to reduce heat loss and the release of epinephrine and norepinephrine from the adrenal medullae, which increase metabolic rate and thus increase heat production (thorough tachycardia, increased vascular resistance and cardiac output). The PMC increases the tone of the skeletal muscle facilitating the activity of the anterior motor neuron, and when the tone increases above a critical level the shivering starts, thus further increasing metabolic rate and heat production. Along with these subconscious mechanisms for body temperature control, a behavioral control of temperature exists. This allows a person to react to cold discomfort by wearing well-insulated clothing or moving around and searching for shelter [3]. When thermoregulatory responses are not enough, core body temperature will drop below 35 °C [4]. A transient organic brain syndrome (termed delirium) may occur in mild hypothermia (core body temperature [Tco] between 32–35 °C) [5]. 

Several aspects of cognition can be affected even by a mild reduction in Tco [6] but contradictory results have been reported regarding which cognitive domains are affected. The effects of cold on cognitive functions was studied using different cognitive tests and different study designs (e.g., duration, modalities, and degree of cooling). Cold exposure was often confounded by other physical and psychological stressors associated with exercise, noise, hypoxia, or environmental conditions (high altitude, deep sea). For example, attention showed either an improvement [7] or a worsening [8] or no effect [9,10] and reasoning was impaired [7,9,11]. Improved driving performance was shown due to the effect of short-term cooling on the perceived sleepiness of car drivers [12], and a short cyclic exposure to extremely low temperature improved cognitive function in patients with mild cognitive impairment [13]. Overall, complex tasks, as compared with simple ones, seem to be more negatively affected by cold exposure and impairment in cognitive performance (CP) seems to be related to the decline in Tco in a dose–response relation [14]. Two major hypotheses can explain these findings. The arousal hypothesis, which states that a slight decrease in Tco drives attention towards the cognitive tasks thus ameliorating performance [15], and the distraction hypothesis, stating that cold stress deviates attention from a primary task [16]. 

The aim of the current review was to systematically evaluate effects of specific cold exposure on cognitive performance in healthy subjects, excluding those related to other confounding factors and clarifying the affected domains, since the impact of cold stress on CP can be relevant to prevent injuries or fatalities in different working categories.

## 2. Materials and Methods

### 2.1. Literature Search on Cold Exposure Effect on Cognitive Performance (Information Sources)

The systematic review was performed following the Preferred Reporting Items for Systematic Reviews and Meta-Analysis (PRISMA) structured guidelines [17].

#### 2.1.1. Information Sources, Search Strategy, and Study Selection 

An extensive systematic literature search on cold effects on cognition was performed by all authors using electronic databases. MEDLINE (through PubMed), EMBASE (Scopus) and PsycINFO were searched using medical subject headings (MeSH) for “cold and cognition” searched in “All Fields” up to June 2021.

The online platform Rayyan [18] was used to select studies based on title and abstract; the selected studies were read thoroughly to identify those suitable for inclusion. Retrieved articles were manually reviewed for potentially eligible citations regarding relevant articles not indexed in the electronic databases. 

#### 2.1.2. Inclusion and Exclusion Criteria 

**Figure 1:**
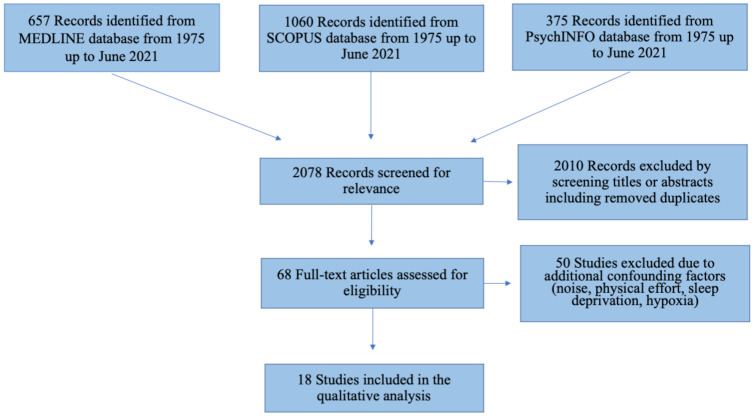
Preferred Reporting Items for Systematic Reviews and Meta-Analyses (PRISMA) flow diagram depicts the number of records identified, included, and excluded, and the reasons for exclusion, through the different phases of the scoping review.

The patients, intervention, comparator, outcomes, and study design (PICOS) approach was used to specify inclusion and exclusion criteria (Table 1). The inclusion criteria were studies with: (1) healthy subjects exposed to a cold environment (either simulated or not) and (2) cognitive performance evaluations related to cold exposure with an experimental design. We excluded: (1) longitudinal studies evaluating CP in different seasons, expert opinions, reviews, comments, letters to the editor, case reports, studies on animals, abstract or conference reports, or articles not written in English; (2) studies where the cold effect was confounded by another variable, such as noise, physical exercise, hypoxia, sleep deprivation, workload, or other stressor factors (e.g., prolonged confinement); (3) studies where the cold was only locally applied and intended to achieve a nociceptive effect; (4) studies involving children; and (5) studies including only electrophysiological cognitive measures (e.g., event related potentials). The primary outcome was to evaluate the effect of cold on cognition. All authors screened the search output, titles, and abstracts in order to find studies matching the predetermined inclusion criteria. The search process and the reasons for exclusion are presented in a flow diagram (Figure 1). A search for duplicate studies was performed.

#### 2.1.3. Data Extraction 

All authors independently extracted the demographic and experimental data from the selected studies (Tables 2 and 3). When disagreement occurred, they reviewed the papers together to reach a consensus. The following data were extracted from each included study: number, gender and mean age of participants, clothes during cold exposure, environmental temperature and duration of the experimental cold exposure, subject temperature and site of measurement, timing of cognitive test (CT) administration (during or after cold exposure), CT type and the related cognitive domains, duration of the test battery, and changes in cognitive performance based on significant results. 

## 3. Results

The literature search retrieved 657 (Pubmed), 1060 (Scopus), and 375 (PsycINFO) articles evaluating the effect of cold exposure on cognitive performance. A total of 68 articles were retrieved for full-text review of which 18 were selected and included based on the inclusion criteria (Figure 1). Studies were published between 1975 and 2021. 

The studies’ results are summarized narratively and presented in Table 2 and Table 3, according to the modalities of simulated cold exposure either in a climate chamber (cold air) or in a laboratory with a water tank (cold water). Most of the studies monitored continuously skin, rectal, or oesophageal temperature.

### 3.1. Climate Chamber Cold Air Exposure Studies (Single vs. Repeated Cold Exposure)

Eight studies investigated the effect of cold air exposure on CP in a climate chamber (see Table 2). All participants except in two studies [10,15] were male and the median age was between 20 and 35 years. Several parameters were different across the studies including the environmental temperature reached (ranging from −10 °C to 10 °C), the time of exposure (ranging from 30 to 120 min) as well as the timing of CT administration (during or after cold exposure). Core body temperature was mostly monitored by a rectal probe, but no major changes were reported; bigger changes were observed by skin temperature (Tsk) measurements. The main cognitive domains evaluated across the studies were executive function, attention and processing speed, memory, and reasoning. 

Overall results showed an impairment of CP in six of the eight studies, while CP was unaffected in two studies after acute cold exposure (ACE). CP was found to be impaired regardless of whether the tests were done in a cold environment or following cold exposure. The only study that specifically investigated both genders in two different experiments showed a partial different response [15]. Yang et al. [19] exposed 6 males for 30 min to cold (−10 °C) and thereafter the participants performed the CT in a warm room, with no difference found in auditory memory (digit span), visual memory (Benton visual retention) and attention (choice reaction time), while a negative effect of ACE was found on manual dexterity, perceptual motor speed (digit symbol test), psychomotor ability (pursuit aiming) and attention/executive function (EF) (Stroop test). Performance in psychomotor ability and perceptual motor speed tasks were decreased and reaction time (RT) for the Stroop word color contradictory task was longer. Muller et al. [20] exposed 10 males to cold (10 °C) for 2 h followed by passive rewarming and participants performed CT multiple times during the study protocol. Attention (choice RT), working memory (WM, digit span backward), short-term memory (STM, digit span forward) and EF explored with the verbal interference task (part I) were found to worsen during both the ACE and the recovery period, while there were no effects on EF with the verbal interference task (part II) and the executive maze task within ACE. Racinais et al. [21] showed different CP in a population of elite skiers compared with healthy controls in CT performed during acute cold (8 °C) exposure. EF was impaired, as shown by a reduction in accuracy during both simple and complex cognitive planning tasks (One Touch Stocking-4 and -6), in healthy subjects but not in elite skiers. Adam et al. [10] exposed 6 males and 2 females to cold (2 °C), but no effect was found on attention (visual vigilance task). Mäkinen et al. [22] also found that acute cold (10 °C) exposure in 10 males induces no effect on CP on simple and complex cognitive tasks involving attention, reasoning, and memory. Watkins et al. [23] reported decreased attentional performance using the numerical vigilance task but not in the dual task after acute cold (−5 °C) exposure in 13 males. Spitznagel et al. [24] observed impairments in attention, WM, and EF in 6 males after acute exposure to cold (10 °C). Enander et al. [15] acutely exposed both 12 male and 12 female participants to cold (around 5 °C). Male participants showed a marked cooling of the extremities and an impairment of manual dexterity tasks, but no impairment in the cognitive domains explored. Despite the shorter cold exposure (60 min vs. 90 min, respectively), females showed an impairment in attention, processing speed and EF, namely an increased number of errors and faster RT for incorrect responses in the digit classification test as well as an increased number of false alarms on the revised color word vigilance task.

**Table 2:** Climate chamber cold air exposure studies.

Authors/ Pubmed ID	Study Protocol: Environmental Temperature (Time of Exposure)/Subject Temperature in Cold	Number of Participants (Gender)/Mean Age of Participants (Mean ± SD)/Clothes	Timing of Cognitive Test Administration/ Duration of Test Battery	Test Battery: Type of Cognitive Test (*Cognitive Domain*)	Changes in Cognitive Performance (Based on Significant Results, *p* < 0.05)
Yang et al. 2021 [19] 33352146	ACE: −10 °C (30 min) vs. 23 °C (30 min)/NA	6 (M)/23.7 ± 1.1 y/ Military uniform (long-sleeved coats, trousers, cotton coats, cotton pants, boots, and hat)	In warm room after environmental exposure/NA	WHO-NCTB: CRT (*attention/response speed*), digit span (*auditory memory*), digit symbol (*perceptual motor speed*), Santa Ana dexterity (*manual dexterity*), Benton visual retention (*visual perception/memory*), pursuit aiming (*motor steadiness*), Stroop (*attention and EF*)	Decreased perceptual motor speed, motor steadiness and attention/EF (Stroop test)
Racinais et al. 2017 [21] 27080805	ACE: 8 °C (30 min) vs. 24 °C (30 min)/Tsk 27.1 °C (~−4.1/skiers) and 24.3 °C (~−6/HC)	36 (M)-22 elite skiers/26 ± 4 y, 14 HC/33 ± 6 y/ Shorts and t-shirts	After 0, 10 or 20 min based on randomization both at 8 and 24 °C/10 min	CANTAB: complex and simple planning task: OTS 6 and 4 (*EF*)	Reduced accuracy in cold only in HC; elite skiers took more time to answer during the test (complex task OTS-6) in cold
Watkins et al. 2014 [23] 25295479	ACE: −5 °C (45 min plus 45 min after a 15 min break) vs. 18 °C (45 min plus 45 min) vs. 30 °C (45 min plus 45 min)/Tsk 24.5 ± 2.6 °C from ~29 °C and Tre 36.9 ± 0.4 °C from ~37 °C	13 (M)/19.6 ± 3 y/ t-shirts, shorts, socks, shoes (same in all session)	Assessed 4 times (0 min; after the first 45 min; just after the break; after additional 45 min)/NA	Numerical vigilance task (*sustained attention),* dual Task performance (*divided attention*)	Sustained attention decreased during cold exposure
Muller et al. 2012 [20] 22506538	ACE: resting at 10 °C (2 h); passive rewarming to 25 °C (2 h) on 3 consecutive days/Tre (~0.2 °C increase) and Tsk (~−9 °C decrease)	10 (M)/23 ± 1 y/ Shorts, socks, gloves (same over the three days). Gloves removed during CT	At baseline (25 °C), after 60 min at 10 °C, after 60 and 300 min after 10 °C exposure/~20 min	IntegNeuro^TM^: CRT (*attention/response speed*), digit span forward and backward (*auditory STM and WM; memory*), verbal Interference part I and II, executive maze task (*EF*)	Both ACE and repeated cold exposure induced decreased CP except EF Maze task and Verbal Interference part II (incongruent task)
Spitznagel et al. 2009 [24] 19653572	ACE: 10 °C (2 h) acute cold exposure at 10 °C; protocol also repeated over 3 consecutive days/Tre and Tsk not reported	6 (M)/23.3 ± 1.5 y/ Shorts, gloves, and socks	13 assessments over the 3 days (at around 4 h- intervals) during and after cold exposure/45 min	IntegNeuro: digit span total (*auditory attention and WM*), CRT (*sustained attention*); verbal interference-word and color-word and mazes (*attention and EF*)	ACE and repeated cold exposure induced CP impairment on attention, WM, and EF
Adam et al. 2008 [10] 18166204	ACE: 2 °C (3 h) vs. 20 °C (3 h) after a pre-exposure to 45 °C (3 h) + room temperature resting period (2 h)/Tre (~0.5 °C increase) and Tsk (~−6–7 °C decrease)	8 (6 M)/24 ± 6 y/ t-shirts, shorts, socks, shoes, cotton gloves and ear band	After ~35 min of cold exposure/20 min	Visual vigilance (*attention*)	No effect on attention
Mäkinen et al. 2006 [22] 16309719	ACE: 25 °C (90 min) followed by 10 °C (120 min); protocol repeated over 10 consecutive days/Tre baseline 37 °C (Δ 0.3−1 °C) and Tsk baseline 26 °C (Δ 6–7 °C)	10 (M)/22.5 ± 1.6 y/ Lightly clad in shorts, socks, and shoes	After 70 min of warm exposure and after 100 min of cold exposure/20 min	ANAM-ICE: digit symbol (*processing speed/sustained attention*), symbol digit modalities test (*sustained attention and WM*); logical reasoning; matching-to-sample (*attention and WM*); continuous performance (*sustained visual attention*); SRT (*attention*); Sternberg memory Search (*visual STM*)	No difference after the first day exposure to cold. Repeated cold exposure caused longer response time and worsening of accuracy and efficiency on sustained attention and WM (symbol digit modalities test); worse accuracy in STM but faster response time in reasoning and sustained attention (continuous performance)
Enander, 1987 [15] 3428250	EXP 1: 2 exp sessions at 5.5 ± 0.5 °C (90 min) vs. 21 ± 0.5 °C (90 min) / Tsk (~−1–3 °C), Tre (~−0.3 °C) EXP 2: 4 ± 1 °C (60 min) vs. 20 ± 0.5 °C (60 min)/Tsk (~−1–3 °C) and Tre (~+0.5 °C)	EXP1:12 (M) office workers/31.4 y (range: 22–45 years)/ Underpants, T-shirt, pants, socks, clogs, and jacket EXP2:12 (F)/ 34.8 y (range: 27–42 years)/ Undergarments, T-shirt, trousers and jacket, socks, and clogs	EXP1: 10 min/55 min EXP2: 35 min/55 min	EXP 1: Color Word Vigilance and SRT (*attention*); Key Tapping and manual dexterity tasks (screw manual dexterity and thumb tapping) EXP2: digit classification (*attention/processing speed*), revised color word vigilance (*attention/EF*), digit addition (*EF*)	EXP1: no effect of cold on attention while manual dexterity was reduced EXP2: effect of cold on attention/processing speed and EF

ACE: acute cold exposure; ANAM-ICE: Automated Neuropsychological Assessment Metric for Isolated and Confined Environments; CANTAB: Cambridge Neuropsychological test Automated Battery; CP: cognitive performance; CRT: choice reaction time; CT: cognitive test; EF: executive function; exp: experiment; HC: healthy control; M: male; NA: not available; OTS: one touch stocking of Cambridge; SD, standard deviation; STM: short term memory; SRT: simple reaction time; T: temperature; Tsk: skin temperature; Tre: rectal temperature; WHO-NCTB: World Health Organization Neurobehavioral Core Test Battery; WM: working memory; y: years.

Three studies investigated CP not only after acute exposure, but also after several days of exposure (up to 10 consecutive days) [20,22,24]. Spitznagel et al. [24] and Muller et al. [20] observed after repeated exposure (three days) to cold an impairment of attention, WM, and EF. Mäkinen et al. [22] found that repeated cold exposure (10 °C) over 10-days causes contradictory effects of both simple and complex cognitive tasks involving attention, reasoning, and memory.

### 3.2. Water Immersion Cold Exposure Studies (Intermittent vs. Continuous Cold Water Immersion and Single vs. Repeated Immersion)

Ten studies investigated the effect of cold-water immersion on CP (see Table 3). Participants of both genders were included in half of the studies and the median age was between 20 and 27 years. Several parameters were different across the studies including water temperature (ranging from ~4.7 °C to 15 °C), the duration of water exposure (ranging from 60 to 180 min), as well as the modality of exposure and timing of CT administration. Intermittent cold-water immersion was used with the same study protocol by Solianik et al. [25,26] and Brazaitis et al. [27] consisting of repeated 20 min periods of cooling followed by 10 min of rest in a room (22 °C) until a rectal temperature (Tre) of 35.5 °C was reached. Half of the studies performed CT during immersion with a different body level of immersion (ranging from lower legs only to head immersed) [8,9,11,28,29] while the remaining half of the studies performed CT after cold water immersion with a quite similar level of body immersion (ranging from chest to only head-out) [7,25,26,27,30]. One study investigated CP during cold air exposure after water immersion [7]. Core body temperature was mostly monitored with a Tco probe (esophageal or rectal) with changes mainly reported up to 35 °C. The main cognitive domains evaluated across the studies are executive function, attention and processing speed, memory, reasoning, and visuospatial abilities. 

**Table 3:** Laboratory cold water immersion studies.

Authors/Pubmed ID	Study Protocol: Environmental Temperature (Time of Exposure)/Subject Temperature in Cold	Number of Participants (Gender)/Mean Age of Participants (Mean ± SD)/Clothes/Immersion of Whole Body or Part	Timing of Cognitive Test Administration/Duration of Test Battery	Test Battery: Type of Cognitive Test (*Cognitive Domain*)	Changes in Cognitive Performance (Based on Significant Results, *p* < 0.05)
Jones et al. 2019 [8] 31047884	7 consecutive (staggered by 24 h) cold water immersion periods at 10 °C (90 min)/Tco (ingestible pill) (~−1.4 °C) and Tsk (~−0.8 to 1.5 °C)	12 (8 M)/26 ± 5 y/ Bathing suit/ Mid-sternum immersion and upright sitting position and leg extended (right arm outside)	Before (25 °C) and during immersion (10 °C) at 5, 30, 60 and 90 min/2 min double-digit addition task; PVT-NA	Double digit addition task (*WM*); PVT (*attention*)	Decrease in both WM and attention performances. After repeated immersion, an improvement in WM
Solianik et al. 2015 [26] 25962329	Intermittent cold water immersion at 14 °C (multiple 20 min of cooling followed by 10 min of rest at 22 °C until Tre reduction to 35.5 °C or 120 min of immersion time)/Tre (−1.1 °C in males and −1.0 in females) and Tsk (−13.7 °C in males and 11.7 °C in females)	27 (14 M)/M 20.6 ± 0.3 y; F 21.0 ± 0.5 y/ Bathing suit/ Semi-recumbent position up to the level of manubrium	Before (22 °C) and 5 min after the end of the cooling procedure (14 °C)/NA	Free-recall test (*memory*); forced-choice recognition memory test (*visual recognition memory*)	Reduced outcome measures on both memory tests only in M related to body cooling
Solianik et al. 2014 [25] 25172303	Intermittent cold water immersion at 14 °C (multiple 20 min of cooling followed by 10 min of rest at 22 °C until Tre reduction to 35.5 °C or 120 min of immersion time)/ Tre (−0.99 ± 0.52 in males and −0.91 ± 0.55 °C in females) and Tsk (−13.53 ± 1.55 in males and −11.91 ± 3.33 °C in females)	32 (18 M)/M 20.7 ± 1.0; F 21.4 ± 2.5/ Bathing suit/ Semi-recumbent position up to the level of manubrium	Before (22 °C) and 5 min after the end of the cooling procedure (14 °C)/NA	Odd/even test (*cognitive flexibility/EF*), forward digit-span (*STM*); forced choice recognition memory (*visual recognition memory*)	Impaired cognitive flexibility (both M and F); impaired visual recognition and forward digit span in M
Brazaitis et al. 2014 [27] 25275647	Intermittent cold water immersion at 14 °C (multiple 20 min of cooling followed by 10 min of rest at 22 °C until Tre reduction to 35.5 °C or 120 min of immersion time)/ Tre (FC: −1.5 °C; SC^2^: −0.9 °C) Tsk (FC: 13.1 ± 1.8 °C; SC^2^: 13.7 ± 1.5 °C)	40 (M); 20 FC group and 20 SC^2^ group / FC 21.2 ± 1.1 y; SC^2^ 22.3 ± 1.7 y/ T-shirt, swim shorts and socks/ Semi-recumbent position with head-out, arms folded across the chest and with legs straight together	Before (22 °C) and 5 min after the end of the cooling procedure (14 °C)/~10 min	Odd/even test (*cognitive flexibility/EF*); forward digit-span task (*STM*); forced-choice recognition memory test (*visual recognition memory*)	Impaired cognitive flexibility; no significant difference between the FC and SC^2^ groups in all the CT
Seo et al. 2013 [28] 24024303	Water tank immersion at 13 ± 1 °C (60 min) or 35 ±1 °C (60 min)/Tsk (Δ −5.8 ± 0.7 °C) and Tre (Δ −0.45 ± 0.2 °C)	9 (M)/23 ± 2 y/ Swimming trunks and a long-sleeved tight-fitting shirt/ Water level up to the iliac crest in a sitting position	Baseline and at 10, 30 and 50 min during cold immersion and 12 min after immersion/~10 min	Stroop Color Word test (SCWT; *attention/EF*)	Impaired SCWT after 13 °C-water immersion
Payne & Cheung, 2007 [29] 17679565	3 sessions with immersion period up to 36 °C Tco at 15° (CC) or 35° (CON) or (SC^1^); after immersion in warm water bath (40 °C) at CC and CON conditions and in SC^1^ seated with only leg immersed in cold water (10 °C)/ Tes was lower during immersion, post-immersion and CT in CC compared with CON and SC^1^ while Tsk was lower in CC during immersion but not during CT compared to SC^1^	12 (M)/23.8 ± 4.5 y/ Clothes:NA/ Immersion up to the neck	After immersion and after ~3 min of either 40 °C or 10 °C-only leg exposure/~10 min	Virtual Hebbs–Williams Mazes (HWM; *visuo-spatial abilities/spatial learning*) Purdue pegboard (*manual dexterity*)	No effect on visuospatial abilities (time to completion and mean error scores) in CC and SC^1^ sessions Effect on manual dexterity
Mahoney et al. 2007 [30] 17585971	10 °C (2 session of 90 min water pool immersion with rewarming period between) adjusted for each participant to induce a Tco drop to 35 °C vs. 35 °C (control condition)/ Tre (~−1.8 °C) and Tsk (~−12 °C)	19 (M+F; not specified)/ 20.5 ± 2.5 y/ Clothes: NA/ Seated and water immersion to the chest (stirred water)	Baseline CT, between the 2 cold (90 min) immersion periods and immediately after immersion/~40 min	Visual vigilance and 4 choice visual RT (*sustained attention*); delayed match-to-sample (DMTS: *WM)*	4 choice reaction time: increased errors and longer RT, match to sample test more errors on delayed paradigm (16 s)
O’Brien et al. 2007 [7] 17078981	Water immersion at 10 °C (adjusted for each participant to induce a Tco drop to 35 °C within 90 min) then rewarming until Tco returns to initial value and then another 90 min in cold water vs. control condition 35 °C/in cold water Tre (−1.5 °C) and Tsk (−11.6 °C)	15 (14 M)/20 ± 2 y/ Clothes: NA/ Chest-deep (arms not immersed) water immersion	After each cooling period and exposure to additional 40 min at 19 °C air tests were performed in a cold room (10 °C)/25 min	U.S. Special Operations Command (SOCOM) subtests: match to sample (*WM*); visual vigilance and complex RT (4 choice) (*sustained attention*), serial addition/subtraction (*attention and WM*), logical reasoning, repeated acquisition *(memory*)	Less correct answers on the match-to-sample, slower response time in addition/subtraction improved response time on vigilance test
Lockhart et al. 2005 [11] 16235879	3 conditions (PDF 1 or 2 or drysuit) in 10 °C stirred water bath (up to 65 min or until core Tes 34 °C)/ Tes at 65 min (drysuit: −0.4 ± 0.2 °C; PDF1: −1.5 ± 0.7 °C; PDF2: −2.8 ± 1.6 °C)	6 (M)/26.8 ± 6 y/ Swimsuit + drysuit or personal flotation device (PFD)/ Drysuit: horizontal positioned with the back of head and chest immersed in the water; PDF 1: semi-recumbent position with head and upper chest outside the water (head-out); PDF 2: horizontal positioned (recumbent position) with the back of head and chest immersed in the water (head-in)	Baseline, immediately after water entry and after 50 min of immersion/ ~15 min	Logic reasoning test; SCWT (*selective attention/EF*); digit symbol coding (*attention/speed of processing*), backward digit span (*WM*); paced auditory serial addition test (PASAT; *auditory attention*)	Increased time to complete SCWT and decreased number of correct responses for digit symbol coding, backward digit span, and the third set of the PASAT with Tes decrease
Baddley et al. 1975 [9] 1205478	Water tank immersion at 4.7 °C (4.4–5.6) vs. 25.8 °C (23.3–26.7) (1 h)/Tre (mean change/drop of 0.72 °C)	14 (NA)/ 23 (range 19–38) y/ Full neoprene wetsuits, bootees, and gloves/Whole body Whole body immersion at a depth of −4.88 m	At the beginning (during) and after each diving/35 to 50 min	Reasoning test; memory test; vigilance test (*attention*)	Impairment on memory; no effect on reasoning and attention

ACE: acute cold exposure; CC: core cooling; CON: immersion period for control; CT: cognitive test; DMTS: delayed match-to-sample; EF: executive function; FC: fast cooling; F: female; M: male; HWM: Hebbs–Williams Mazes; NA: not available; PASAT: paced auditory serial addition task; PDF: personal flotation device; PVT: psychomotor vigilance task; RT: reaction time; SC^1^: superficial cooling; SC^2^: slow cooling; SCWT: Stroop Color Word test; SD, standard deviation; STM: short-term memory; T: temperature; Tco: core body temperature; Tre: rectal temperature; Tsk: skin temperature; Tes: oesophageal temperature; SOCOM: U.S. Special Operations Command; WM: working memory; y: years.

Overall results show an impairment of CP in nine of the ten studies. CP impairment was found when tests were completed both during and after water immersion. Two studies performed by the same group show partially different results for males and females [25,26]. Solianik et al. [25,26] showed that intermittent whole-body immersion in cold water (14 °C) affects CP mostly in men despite the fact that the cooling rate did not differ between genders. Another study with a similar cooling procedure [27] but with different clothes (and only male) did not find any changes in memory while confirmed the impairment of cognitive flexibility showed by Solianik et al. [25] on both genders. Moreover, Brazaitis et al. [27] did not find any difference between individuals exhibiting a fast or slow cooling rate (i.e., decrease in Tre). Jones et al. [8] found that acute exposure to cold water (10 °C) impaired both WM and attention, while repeated immersion (cold acclimatization) led to an improvement in WM but not in attention. Baddeley et al. [9] exposed 14 divers wearing a neoprene suit to 1-h of cold (~4.7 °C) (under)water tank immersion and showed that reasoning and attention were not affected while memory was affected (cognitive test performed during immersion). In the study of Seo et al. [28] scores related to the Stroop color word test (word, color, word-color, and interference) measuring the selective attention and the ability to suppress preprogrammed responses (cognitive inhibition) did not change during immersion in cold (13 °C) water but were significantly impaired during the post-immersion recovery. Stroop performance impairments correlated with the increased metabolic rate. Lockhart et al. [11] addressed cooling in water (10 °C) on three different conditions. Immersion of the back of the head and upper chest increased the speed of hypothermia onset during cold water immersion and oesophageal temperature (Tes) decrement was correlated with a worsening in selective and auditory attention, WM, and speed of processing. Mahoney et al. [30] exposed participants to 10 °C water immersion and found that cold exposure impaired both attention (slowing of RT and increased errors in the 4 choice RT task) and WM performance (delayed match-to-sample, increased errors in the longest delay interval (16 s)). O’Brien et al. [7] exposed participants to 10 °C water immersion and found that, even after 40 min following the end of water immersion, in cold air (10 °C) there was an impairment of WM in the match-to-sample test and that response time was slower in the addition/subtraction test. There was an improvement in the visual vigilance test assessing sustained visual attention.

Only Payne & Cheung [29] did not find any changes in CP when evaluating the effect of core cooling without any skin cooling/distraction influence on visuospatial abilities and manual dexterity. There was no effect of core cooling or superficial leg cooling on time to completion or maze errors in a computerized version of the Hebb–Williams mazes. 

Only two studies found an improvement in CP, specifically on attention [7] and WM [8] but the latter found the improvement not after ACE but after repeated exposure. 

## 4. Discussion

The main findings of the current systematic review are that acute cold exposure induced an impairment of CP (in 15 of the 18 studies) in most of the experimental settings even in healthy subjects in a simulated setting and before accidental hypothermia was established. The most investigated and affected cognitive domains were attention and processing speed, executive function, and memory. Processing speed and executive function showed an impairment, while memory and attention showed contrasting results. Impairment of CP was observed both during exposure to cold air and cold water and persisted even after exposure and during passive re-warming. The impact of cold water exposure seems to be more pronounced than that of cold air and correlated to a clearer core body temperature drop. Though the more prevalent finding was of an impairment of CP following acute cold exposures, one single study also showed an improvement in an individual cognitive domain (attention) [7]. There were only limited data on possible gender differences, but males and females seem to have a different response to cold exposure even in CP. Repeated exposure and possible acclimation effect also seem to affect CP, but further studies are needed to further clarify the current findings. 

### 4.1. Cognitive Performances and Differences between Studies

The majority of the studies analyzed in this review show that a single acute exposure to cold (either cold air or cold water) impairs CP (attention, memory, executive function and speed of processing) and that the extent of these effects depends mainly on individual physiological responses to cold but also on the extent of the exposure in terms of duration and Tco reached. Several discrepancies that require discussion can be observed. Given the different effect of cold air versus cold water exposure, the studies investigating CP after exposure to cold air or cold water are described separately. 

#### 4.1.1. Climate Chamber Cold Air Exposure Studies

Overall, cold air exposure was shown to impair some cognitive domains such as attention and speed of processing [15,19,20,23,24], memory [20,24], and executive function [15,19,20,21] while no effect has been shown in reasoning [22]. In contrast, some studies report no effect on attention [10,15,22] and memory [19,22]. The contrasting results on memory and attention can be related to different aspects, such as the length and temperature of cold exposure, the timing of execution of CT (during or after cold exposure), different CT exploring the same cognitive domain, and the clothes worn by the participants. Studies with more severe environmental conditions and with an adequate sample size generally show an effect of cold on CP. Yang et al. [19] and Mäkinen et al. [22] evaluated attention/processing speed using the digit symbol test showing an opposite effect that may be related to several differences such as environmental temperature (−10 °C vs. 10 °C), the length of cold exposure (30 vs. 120 min), and the timing of CT execution (after and during cold exposure). Opposite effects on attention are also shown by Watkins [23] and Adam [10] using the same test (visual vigilance). Both used different environmental temperatures (−5 °C vs. 2 °C) and different protocol lengths (90 vs. 120 min); both performed the CT during the exposure. The use of different cognitive tests as well as the timing of their administration also brought different results. Attention was not affected when measured with simple RT [10,15], but was affected when measured with choice RT [20]. Muller et al. [20] and Spitznagel et al. [24] showed an impairment on memory employing the digit span to investigate both STM and WM, while Mäkinen et al. [22] and Yang et al. [19] did not report any impairment using different tests (match-to-sample, Sternberg memory search and Benton visual retention). 

Three studies investigated the CP not only after acute exposure but also after repeated exposures, addressing the possible effect of cold acclimation [20,22,24]. Specifically, Muller et al. [20] exposed the participants to cold on three consecutive days (2 h each day) similarly to Spitznagel et al. [24]. Both showed a CP impairment (memory, attention, and EF). Mäkinen et al. [22] exposed participants to the same setting up to 10 consecutive days and showed an impairment on accuracy in the sustained attention/WM (symbol digit modalities test) and visual STM (Stenberg memory search), as well as on efficiency and response time in attention/WM (symbol digit modalities test) but also showed an improvement in the logical reasoning task and sustained visual attention (continuous performance test). Muller et al. [20] found that CP was reduced both during cold exposure and during the rewarming phase despite the physiological values (e.g., temperature) returning to baseline levels during the rewarming phase; both the distraction theory and the arousal hypothesis could not explain these findings, but authors hypothesized that possible acute brain vascular changes (e.g., vasoconstriction) could provoke the cognitive dysfunctions [31]. 

#### 4.1.2. Water Immersion Cold Exposure Studies

Overall, cold water immersion exposure impaired some cognitive domains such as attention [8,11,28,30], executive function [25,27], memory [7,8,9,11,25,26,30], and speed of processing [11] while no effect was shown in visuospatial abilities [29] and reasoning [7,9,11]. Similarly, no effect on attention [7,9] and memory [27] have been reported. The contrasting results of cold water immersion on attention and memory can be related to different aspects, as discussed above for those studies regarding cold air environments, such as the length and water-temperature during the cold exposure, the timing of execution of CT (during or after cold water immersion), different CT exploring the same cognitive domain, different protocol (intermittent vs. continuous cold water immersion and repeated exposure), whether the whole body or different body parts were immersed (e.g., iliac crest, lower legs, sternum), and previous cold acclimation. Memory has been investigated by different tests in most of the studies; while the majority found an effect of cold on memory, Brazaitis et al. [27] found no effect despite employing the same two CT to evaluate memory (forward digit span and forced choice recognition memory) used by Solianik et al. [25] and (forced choice recognition memory) by Solianik et al. [26]. The only difference in those studies was the body level of immersion in the water and the worn clothes. One potential explanation of the different results could be that participants in the study of Brazaitis et al. [27] were recruited from a larger study on cold exposure tolerance and that previous acclimation may have had an impact. Attention was explored mostly with different tests and mainly reported to be affected by cold exposure. O’Brien et al. [7] and Baddeley et al. [9] did not find a significant effect on attention, despite O’Brien employed the same tests and a comparable study protocol as Mahoney et al. [30]. Baddeley et al. [9] employed a population of divers who were fully and continuously immersed underwater and the CT were performed during (and not after) immersion. 

Only Jones et al. [8] explored the effect of cold acclimation in water [8]. Participants were repeatedly immersed in cold water for 7 days and showed that insulative cold acclimation occurred. Attention (evaluated with the psychomotor vigilance task) worsened during all immersions suggesting no effects of cold acclimation, while WM improved after repeated immersions. Previous studies show that thermal adaptation can improve thermal sensation and comfort [32]. Cold acclimation manifests with different physiological mechanisms such as a reduction in shivering, and vasoconstriction response (habituation), generating (metabolic response) and retaining (insulative response) heat [32]. The first response appears in the first two days after cold exposure and depends on the type and intensity of cold. Habituation of thermal sensation is caused by reduced vasoconstriction and blood pressure, higher Tsk, delayed onset and reduced intensity of shivering, reduction in stress hormones released, and less intense sensations of cold and thermal discomfort. Habituation has been shown to be mediated by central pathways rather than peripheral [33]. Metabolic acclimation is related to increased metabolic rate thus increasing heat generation through shivering and non-shivering thermogenesis. The insulative cold acclimation is determined by an enhanced vasoconstriction that reduces heat loss and thus decreases Tsk, delaying the onset of shivering and reducing energy lost for heat generation by muscle activity. 

### 4.2. Evidence and Existing Theories

Cold exposure results in whole-body cooling that may reduce Tco. Cooling is enhanced by exposure to cold water or wind, which both increase convective heat loss. A brief (<30 min) or intermittent exposure to cold (water immersion or air) with low thermal protection induces peripheral cooling (reduced Tsk, increased sympathetic response, and discomfort) while a prolonged exposure induces whole body cooling with lower Tsk and Tco, thus also reducing brain temperature. Cold exposure can lower Tsk and induce mild shivering without reducing Tco which may result in cognitive distraction due to abnormal thermal sensation. Distraction caused by cold discomfort can be demanding for central attention resources and thus decrease CP. Teichner [16] proposed the distraction hypothesis where cold stress is considered the cause of a deviation of attention away from the primary task provoking an impairment of performance such as an increased number of missing signals and a slowing of RT rather than rapid mistakes [16]. All studies included in the present review were performed in a simulated environment, allowing a control of confounding factors compared to in-field studies. Cold exposure in a water environment more commonly induced a reduction of Tco and Tsk and, in a parallel manner, 9 out of 10 studies showed an impairment in CP; cold exposure to air did not induce significant alteration to Tco, even if Tsk were mostly affected and 6 out of 8 studies showed a CP impairment. Solianik et al. [26] showed an impairment on memory in male subjects and higher levels of epinephrine and cortisol that could support an additional negative effect of stress. One study assessed thermal comfort and sensation and shows an impairment after ACE, but a CP impairment was found only in the control population compared with the elite skiers [21]. The authors speculate that since elite skiers usually train in cold environments, they may be able to maintain focused attention on the task. Another study performed in cold water did not show an additional distracting effect of superficial cooling (legs in 10 °C while performing CT) compared with a control setting where people were immersed in a warm water bath (40 °C) [29]. 

One out of 18 studies show an improvement in one cognitive domain (attention) after ACE [7]. The arousal hypothesis proposes that the effects of Tco decline is experienced as a challenge by the body. This may initially lead to an improved performance which will subsequently decline as the cooling persists. Such improvement in CP has been related to increased sympathetic activity [15]. The theory states that the degree of stimulation related to task difficulty and subject experience may explain the findings of improved performance [15]. Despite the drop in Tco and the increase in sympathetic response, CP was found impaired in male subjects in the Solianik et al. study [26]. Different study protocols in terms of environmental exposure and timing of CT administration may partially explain the disagreement of most of the study findings with such hypothesis, despite the fact that an overall impairment was confirmed when CT were administered both during acute cold exposure and after the recovery period.

### 4.3. Practical Implications and Future Research Perspectives

It is evident from this review that several factors, such as the severity and the duration of the exposure to cold, are relevant to reduce CP. Such impairment mainly involves more complex cognitive tasks. The negative impact of cold stress on CP is relevant since different working categories (e.g., fishermen, mountain rescuers, soldiers) are exposed to different levels of cold and the cognitive effect of cold may result in injuries or fatalities. The findings of the current review may help to better recognize when such CP impairment may be expected and also to develop new strategies to reduce it in a gender-oriented way. 

Clothing for protection from the cold, tyrosine supplementation [7,30] or cold acclimation have been suggested to reduce cognitive impairment induced by cold. The aim of these strategies is to reduce thermal discomfort and physiological and psychological distress allowing for the maintenance of focused attention on a specific task. Mäkinen et al. [22] and Jones et al. [8] showed that repeated cold exposure over several days can improve thermal sensation reducing cold discomfort and lead to improvement in some CP, even while other cognitive domains seem to remain impaired [22]. Other current literature findings are contradictory [20,22,24]. Further studies should be performed to clarify possible positive/negative effects of different cold-acclimation strategies. 

## 5. Conclusions

The main findings of the current systematic review are that in most of the experimental settings (either cold air or cold water) cold exposure induced an impairment of CP even in healthy participants and before accidental hypothermia was established. The majority of the studies show that a single acute exposure to cold may impair attention, speed of processing, memory and executive function and these effects might depend on individual physiological responses to cold as well as the extent of the exposure in terms of duration and temperature reached. Despite the fact that data are scarce, males and females seem to have a different response to cold exposure even in CP. Repeated exposure and possible acclimation effect seems also to have an impact on CP, but further studies are needed to confirm such preliminary findings.

## Figures and Tables

**Table 1:** Patients, intervention, comparator, outcomes, and study (PICOS) design criteria for inclusion and exclusion of studies.

Parameter	Inclusion Criteria	Exclusion Criteria
**Subjects**	Healthy adults undergoing cognitive tests under simulated or unsimulated cold exposure (acute or repeated cold exposure) Age > 18 years Any gender	Age < 18 years Cold locally applied to achieve a nociceptive effect Studies including only electrophysiological cognitive measures
**Intervention**	Simulated cold exposure (cold air or cold water immersion)	—
**Comparator**	Warm air or water exposure	—
**Outcomes**	Effect of cold exposure on cognitive performance	—
**Study design**	Interventional, cross-sectional studies published in English	Longitudinal studies, reviews, expert opinions, comments, letter to editor, case reports, studies on animals, abstract and conference reports. Published in any other language than English.

## Data Availability

The data that support the findings of this study are available from the corresponding author, upon reasonable request.
